# Designing healthy communities: creating evidence on metrics for built environment features associated with walkable neighbourhood activity centres

**DOI:** 10.1186/s12966-017-0621-9

**Published:** 2017-12-04

**Authors:** Lucy Dubrelle Gunn, Suzanne Mavoa, Claire Boulangé, Paula Hooper, Anne Kavanagh, Billie Giles-Corti

**Affiliations:** 1 Healthy Liveable Cities Group, Centre for Urban Research, College for Design and Social Context, Royal Melbourne Institute of Technology, Melbourne, Victoria 3010 Australia; 2 Noncommunicable Disease Unit, Centre for Health Equity, Melbourne School of Population and Global Health, The University of Melbourne, Melbourne, Victoria Australia; 3 Centre for the Built Environment and Health, School of Earth and Environment and School of Sports Science, Exercise & Health, University of Western Australia, Perth, Western Australia Australia; 4 Gender and Women’s Health Unit, Centre for Health Equity, Melbourne School of Population and Global Health, The University of Melbourne, Melbourne, Victoria Australia

**Keywords:** Transport walking, Planning policy, Built environment, Urban design, Neighbourhood activity/town centre, Cluster analysis, Land use mix, Geographic information systems

## Abstract

**Background:**

Evidence-based metrics are needed to inform urban policy to create healthy walkable communities. Most active living research has developed metrics of the environment around residential addresses, ignoring other important walking locations. Therefore, this study examined: metrics for built environment features surrounding local shopping centres, (known in Melbourne, Australia as neighbourhood activity centres (NACs) which are typically anchored by a supermarket); the association between NACs and transport walking; and, policy compliance for supermarket provision.

**Methods:**

In this observational study, cluster analysis was used to categorize 534 NACs in Melbourne, Australia by their built environment features. The NACS were linked to eligible Victorian Integrated Survey of Travel Activity 2009-2010 (VISTA) survey participants (*n*=19,984). Adjusted multilevel logistic regressions estimated associations between each cluster typology and two outcomes of daily walking: any transport walking; and, any ‘neighbourhood’ transport walking. Distance between residential dwellings and closest NAC was assessed to evaluate compliance with local planning policy on supermarket locations.

**Results:**

Metrics for 19 built environment features were estimated and three NAC clusters associated with walkability were identified. NACs with significantly higher street connectivity (mean:161, SD:20), destination diversity (mean:16, SD:0.4); and net residential density (mean:77, SD:65) were interpreted as being ‘highly walkable’ when compared with ‘low walkable’ NACs, which had lower street connectivity (mean:57, SD:15); destination diversity (mean:11, SD:3); and net residential density (mean:10, SD:3). The odds of any daily transport walking was 5.85 times higher (95% CI: 4.22, 8.11), and for any ‘neighborhood’ transport walking 8.66 (95% CI: 5.89, 12.72) times higher, for residents whose closest NAC was highly walkable compared with those living near low walkable NACs. Only highly walkable NACs met the policy requirement that residents live within 1km of a local supermarket.

**Conclusions:**

Built environment features surrounding NACs must reach certain levels to encourage walking and deliver walkable communities. Research and metrics about the type and quantity of built environment features around both walking trip origins and destinations is needed to inform urban planning policies and urban design guidelines.

**Electronic supplementary material:**

The online version of this article (10.1186/s12966-017-0621-9) contains supplementary material, which is available to authorized users.

## Background

Active transport – including walking for transport– [1–4] is both health-promoting and supports sustainable living [5, 6]. A growing body of international evidence [7–10], shows the built environment plays an important role in creating pedestrian-friendly neighbourhoods that promote walking and reduce chronic disease risk factors.

A number of built environment features are consistently shown to facilitate transport walking around residential homes, which are the origins of many walking trips. These include: highly connected streets, high population density, mixed land use and good access to destinations and transit, and sidewalk provision [11–15]. However, to inform planning policy and urban design guidelines, policy-makers and urban designers require specific information on the types, quantities and mix of built environment features that influence walking [3, 16–21]. Moreover, most of the research to date has focused on the home environment [22], with less research focused on the end points of those trips.

Of all the built environment features that promote walking, it is destinations that are most important because they provide a ‘reason to walk’ and an end-point for walking trips [23]. Destinations and destination diversity are repeatedly shown in active living research studies to be positively associated with transport walking [10, 24–29]. Yet, unlike transport academics [30], active living researchers rarely study the built environment features surrounding destinations when undertaking travel mode studies.

In local communities, destinations are often co-located and concentrated at local shopping centres, known in Melbourne, Australia as ‘neighbourhood activity centres’ (NACs) [25, 31, 32]. NACs function as a community focal point and enable local living by providing a variety of retail shops, services and destinations. Their distribution across cities are important because they facilitate the creation of the ‘20- (or 30-minute) city,’ a concept that is drawing growing global interest [32–34]. However, to attract pedestrians the built environment surrounding NACs must support walking [33, 35, 36].

Understanding the type, quantities and mix of built environment features and their influence on walking trips is therefore crucial for designing and retrofitting NACs to be pedestrian-friendly. One method to evaluate this is cluster analysis, which groups built environment features to help identify walkable neighbourhood environments [3, 4, 25, 36–41]. Several authors have modeled associations between cluster type (treated as an exposure variable) and walking, and have found that more walkable neighbourhoods (as defined by cluster analysis) are typically associated with increased odds of walking [3, 4, 25]. Most research using cluster analysis has focused on home neighbourhoods [4, 25, 37, 40], with few studies, if any, providing metrics on the type, quantities and mix of built environment features that encourage walking or consider destinations, such as NACs, that could inform relevant urban design guidelines [36].

In Melbourne, Australia, precinct structure plans typically allow for town centres or NACs to be anchored by supermarkets. Furthermore, Melbourne’s planning policy stipulates that “80-90% of households should be within 1km of a town centre of sufficient size to allow for provision of a supermarket” [42]. However, unless policy implementation is evaluated and measured on the ground [43] it remains unclear whether this policy requirement is being met. Indeed, few studies measure urban design policy implementation in Australia [3, 44]. Nevertheless, this type of evidence is useful to assess whether NACs have the potential to facilitate transport walking [45]; and, could help achieve the desired 20-minute city in Melbourne.

The aims of this paper were to: (1) investigate the type, quantity and mix of a broad range of built environment features to create a walkability typology of NACs across Melbourne, Australia; (2) examine whether built environment features surrounding NACs are associated with local residents’ transport walking; (3) assess the spatial distribution of NACs in relation to walkability; and (4) assess compliance with local planning policy about the location of supermarkets in relation to residential dwellings.

## Methods

### Individual-level data

Individual level data from the Victorian Integrated Survey of Travel Activity (VISTA) 2009-2010 were used [46]. VISTA is a repeat cross-sectional survey of one-day travel behavior administered by the Victorian Department of Economic Development, Jobs, Transport, and Resources.

Data for urban areas of metropolitan Melbourne were extracted for this study for 21,664 adults aged 18 years and over. VISTA follows a multi-stage stratified sampling process. First, a stratified random sample was taken across eight regions in Melbourne, and within each region several Census Collector Districts (CCD) were selected followed by sampling individual households within CCDs. CCDs cover an average of 225 dwellings. Due to low response rates in some areas, additional households were selected to meet response rate requirements. The survey asks questions on one day of travel behavior for each individual within each household. Further information on the VISTA survey can be found in online Additional file 1: Appendix 1 VISTA Sample.

### Activity centre data

The NAC dataset was defined as all commercially zoned property boundaries within an 800m street network buffer of selected supermarkets. An 800m buffer distance was used here, since several researchers have found associations between built environment correlates and physical activity measures at this distance [47–49]. In particular, Manaugh et al (2011) found associations with Walkscore and a Walkability Index and home-based shopping trips at 800m [49] and Gunn et al (2017) found increased odds of transport walking for those located within 800m of local food outlets including supermarkets, cafés/takeaway stores, and small food stores, which are typically co-located within NACs [48].

Property data were sourced from VicMap Property [50]. Street centrelines were sourced from VicMap Transport [51] and freeways and freeway on-ramps were removed to create a walkable road dataset from which the 800m street network buffers around NACs were created.

Geocoded supermarket data were obtained from a commercial provider [52] and spatially mapped using Arc GIS 10.2. This data set was validated against the state’s strategic planning framework which identifies major NACs across Melbourne (i.e., *Plan Melbourne* [33]) through which a number of additional supermarkets were identified and manually geocoded*.* Data for commercial zones were obtained from VicMap Planning [50]. This was overlaid onto the supermarket layer, to ensure that identified supermarkets were associated with commercial zones with other retail stores. Manual checking of the NAC layer was conducted using Google Maps. Supermarkets not placed within a commercial zone were removed from the dataset. Since many NACs contain more than one supermarket, duplicate supermarket addresses and duplicate NACs were removed from the dataset, however information on how many different supermarkets existed in each NAC was retained as a supermarket diversity variable.

### Dependent variables

**Fig. 1:**
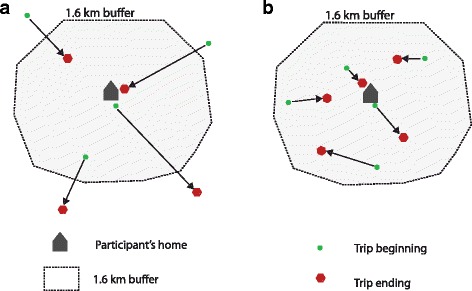
Dependent variable definitions for transport walking trips. **a** Any transport walking. **b** Any neighbourhood transport walking

Two dependent variables were developed using VISTA daily transport walking trip data. First, due to the non-normal distribution of VISTA walking data, *any transport walking* was defined as all daily transport walking trips dichotomized into ‘one or more walking trips’ and ‘no walking trips’[44, 53]. Second, to capture local transport walking, *any neighbourhood transport walking* was defined as daily walking trips beginning and ending within a 1.6km street network buffer of a participants’ home. This data was dichotomized into ‘one or more walking trips within the neighbourhood’ and ‘no walking trips in the neighbourhood’ (see Fig. 1).

### Independent variables

#### Built environment

Following the work of Hooper et al (2014), metrics for 19 built environment features were defined for three domains: community design, movement network, and lot layout [43].

Community design was measured using the presence and quantity of a variety of destinations with accessibility to these destinations measured using the number of transport stops and pedshed ratios (i.e.,the ratio of area within 800m street network buffer to the area within 800m Euclidean buffer). Based on planning guidelines, Hooper et al (2014) also define the movement network made up of design features such as street connectivity, block perimeters and the number of cul de sacs [54] which determines the directness of pedestrian routes and the proximity of local destinations. The lot layout domain includes housing diversity and net residential density [43], which determines the number of residents living in close proximity to NACs and hence, supports the viability of retail shops and transit services. Built environment features were calculated within an 800m street network buffer of the NAC using GIS methods and ArcGIS 10.2 software (see Table 1).

**Table 1:** Built environment variables calculated within 800m of a supermarket

Variables and definitions
*Community Design*
Pedshed: ratio of area within 800m street network buffer to the area within 800m Euclidean buffer
Number of community resources: post offices, community centres, child care centres, libraries
Number of small food stores: butcher, green grocers, convenience stores
Number of other retail stores: banks, pharmacy, petrol station, newsagent
Number of supermarkets: includes major and minor supermarkets
Supermarket diversity: number of different major supermarkets (0-4)
Number of transport stops: buses, trams, train
Transport diversity: number of different types of transport (0-5)
Destination diversity: number of different individual destination types (0-16)
*Movement Network*
Street connectivity: number of ≥3 way intersections
Cul de sacs: number of cul de sacs
Cul de sac segments ≤120m long: number of cul de sac segments ≤120m long
Connected node ratio: number of ≥3 way intersections ÷ all intersections including cul de sacs
Disconnected node ratio: number of cul de sacs ÷ all intersections including cul de sacs
Mean block perimeter (m)
Walkable block ratio: number of blocks ≤620m perimeter ÷ total number of blocks
Traffic exposure ratio: length of low traffic roads ÷ length of low and high traffic roads
*Lot Layout*
Housing diversity: number of different housing types (0-8)
Net residential density: number of commercial dwellings + number of residential dwellings ÷ commercial and residential area

#### Confounders

A number of confounders likely to affect transport walking trips undertaken by participants were identified from the literature including: age, sex, studying (yes, no), occupation (unemployed, casual work, part-time, full-time), household type (couple no children, couple with children, other household structure including single parent, sole person, and other household structure undefined), income (<$650, $650-$1099, $1200-$1949, $1950-$2499, $2500), own a motor vehicle (do not own a motor vehicle, own a motor vehicle), and area level disadvantage derived from the Australian Bureau of Statistics Socio-Economic Index for Areas, Index of Relative Social Disadvantage (SEIFA IRSD) based on deciles (high: 1-4, medium: 5-7, low: 8-10) [44].

### Statistical analysis

#### Cluster analysis of neighbourhood activity centres

Cluster analysis creates homogenous clusters of built environment variables whilst ensuring that each cluster is distinct from the others [55]. It was used to identify NAC typologies based on the built environment features listed in Table 1.

Specifically, a two-stage cluster analysis was undertaken. K-means clustering was used since it performs better in the presence of outliers and irrelevant clustering variables [55], however the number of clusters must be specified *a priori.* To help establish this, in the first stage, hierarchical clustering and the Calinski-Harabasz pseudo F statistic were estimated. The number of clusters was identified from large changes in the error coefficients between stages in the agglomeration schedule of the hierarchical cluster analysis, and from identifying the largest Calinski-Harabasz pseudo F statistic for each cluster solution.

The final K-means cluster solution was chosen based on interpretability of the average values of the built environment features. This was undertaken to identify cluster types relating to walkability. The final NAC cluster solution was mapped to examine the spatial location of the NAC cluster typologies across metropolitan Melbourne.

ANOVA and Tukey post-hoc tests were undertaken on the final K-means cluster solution to establish whether there were significant differences for each built environment feature by cluster type. Results are presented as a cross tabulation of the built environment features by cluster type. The tests and the cross tabulation cluster solution indicate which built environment features are different between NAC cluster types and by ‘how much’. This information is useful for developing and monitoring evidence-based policy and design standards for new urban areas and NACs, and for retrofitting NAC areas to increase walkability.

All clustering was undertaken on standardized built environment features to remove the effect of differing scales.

#### Measuring associations with transport walking trips and NAC cluster types

Several participants had no measure of area level disadvantage (*n*=155) and a further 7% of the sample were not associated with a NAC due to living on the extreme periphery of the city. Participants with these observations were removed from the sample with all remaining analyses conducted on complete cases within urban areas of Greater Melbourne on a sample of 19,984 observations. Cross tabulations between participant’s individual level variables and the NAC cluster types were undertaken.

Multilevel logistic regression models were estimated to measure associations between the NAC cluster type included as dummy variables with the LW NACs as the reference, and any transport and any neighbourhood transport walking trips respectively. Models were adjusted for confounders and allowed for clustering of individuals within households within the geography of statistical area level 2 (SA2). CCDs are clustered within SA2 areas. SA2 areas typically cover an average population of 10,000 people and allow sufficient coverage of clustered households and their 1.6km street network buffers. Odds ratios and 95% confidence intervals are reported. All analyses were undertaken using SPSS and Stata IC 13.0.

#### Measuring policy implementation of NAC access

A descriptive analysis was undertaken to understand NAC access of VISTA survey participants. The closest NAC was identified for each participant’s residential location using the street network. The distance to the closest NAC and the percentage of participants who lived within various threshold distances were calculated. The first distance threshold of 1km was based on current policy guidelines in Victoria that stipulate that “80-90% of households should be within 1km of a town centre of sufficient size to allow for provision of a supermarket” [42]. The second distance threshold was based on recent empirical findings suggesting that locating local food outlets at distances of up to 800m may influence transport walking trips [48]. The third threshold distance of 1.6km was based on a commonly used distance for determining walkable neighbourhoods which is said to be associated with achieving a brisk 15 minute walk [4, 56].

## Results

### Cluster analysis

**Fig. 2:**
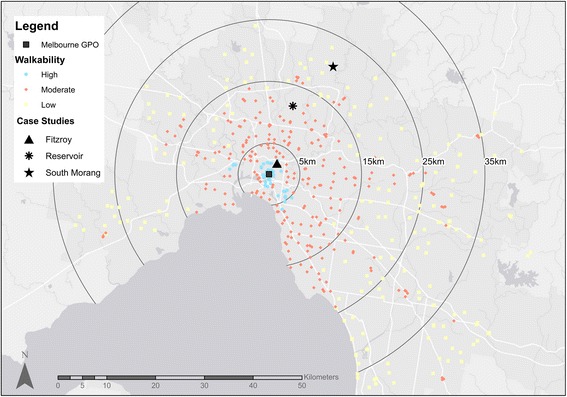
Map of metropolitan Melbourne with neighbourhood activity centres (NACs) displayed by walkability

Results from the hierarchical cluster analysis confirmed that three clusters were appropriate for the NAC data. Table 2 and Fig. 2 present summary statistics and a map for the three cluster solution respectively.

**Table 2:** Descriptive Statistics for the three cluster solution for NACs

*Cluster:*	Overall: *n*=534		High (HW) : *n*=48		Moderate (MW): *n*=267		Low (LW): *n*=219	
*Built Environment Variables*	Mean	SD^a^	Mean	SD	Mean	SD	Mean	SD
*Community Design*								
Pedshed	0.53	0.10	0.63 ^**^	0.04	0.57 ^**^	0.07	0.46^**^	0.09
Community resources	11.03	7.75	27.19 ^**^	6.28	12.42 ^**^	6.06	5.79 ^**^	2.47
Small food stores	16.54	18.63	62.63 ^**^	22.32	17.55 ^**^	10.44	5.20 ^**^	3.19
Other retail	21.37	32.33	101.77 ^**^	60.36	18.97 ^**^	9.23	6.67 ^**^	4.35
Supermarkets	3.64	3.53	12.08 ^**^	5.53	3.55 ^**^	1.79	1.89 ^**^	1.04
Supermarket diversity	1.17	0.47	1.02	0.14	1.11	0.38	1.26 ^**^	0.59
Transport stops	70.22	48.92	183.52 ^**^	61.00	76.51 ^**^	26.18	37.71 ^**^	16.59
Transport diversity	2.13	1.04	3.71 ^**^	1.05	2.44^**^	0.84	1.40 ^**^	0.56
Destination diversity	12.80	2.92	15.85 ^**^	0.36	14.14 ^**^	1.63	10.49 ^**^	2.82
*Movement Network*								
Street connectivity	73.74	33.94	160.77 ^**^	20.20	71.66 ^**^	21.59	57.21 ^**^	14.54
Cul de sacs	78.43	60.02	217.38 ^**^	75.74	51.30 ^**^	25.66	81.06 ^**^	40.09
Cul de sac segments≤120m long	64.53	59.15	210.19 ^**^	74.75	40.70 ^**^	23.70	61.66 ^**^	35.91
Connected node ratio	0.19	0.08	0.21 ^**^	0.05	0.13 ^**^	0.04	0.27 ^**^	0.06
Disconnected node ratio	1.05	0.52	1.34	0.39	0.71 ^**^	0.27	1.39	0.52
Mean block perimeter	1428.44	1023.18	691.20	218.72	941.74	381.67	2183.40 ^**^	1178.63
Walkable block ratio	0.52	0.20	0.78 ^**^	0.05	0.60 ^**^	0.14	0.37 ^**^	0.15
Traffic exposure ratio	0.81	0.08	0.73 ^**^	0.07	0.81	0.07	0.83	0.08
*Lot Layout*								
Housing diversity	6.55	1.55	6.48	1.66	6.54	1.55	6.57	1.52
Net residential density	20.96	28.55	76.99^**^	65.31	19.82 ^**^	14.38	10.07 ^**^	2.97

^** ^Significantly different between remaining cluster types based on Tukey post-hoc tests with *p*<0.01

^a^ *SD* Standard deviation

The first cluster identified had built environment features that were more pedestrian-friendly or ‘highly walkable’ (HW) in terms of community design (i.e., higher pedshed ratios, higher mean values for all destinations, and higher transport and destination diversity scores); the movement network (i.e., higher street connectivity, higher walkable block ratios) and lot layout (i.e., higher net residential densities). Despite being HW, these NACs had higher mean numbers of cul de sacs, and cul de sac segments≤120m long and almost no supermarket diversity.

The second cluster characterized ‘moderately walkable’ NACs (MW) with moderate values for most community design, movement network and lot layout domains. The MW NACs had the lowest values for cul de sacs and cul de sac segments≤120m long, connected and disconnected node ratios. However, destination diversity near MW NACs remained high and was similar to the HW NACs; and, they also had similar mean values to the HW NACs for the mean block perimeter. Traffic exposure ratios in MW NACs were similar to the final cluster.

The final cluster was characterized as ‘low walkable’ (LW) because NACs in this cluster had the lowest mean values for most of the three aforementioned domains. Table 2 shows that the low walkability of these NACs was also reflected in some of the remaining movement network variables (i.e., high cul de sacs, cul de sac segments ≤120m long, large mean block perimeter, and high traffic exposure ratio). Conversely, the LW NACs had the highest mean values for connected node ratios, but a similar mean value to HW NACs for disconnected node ratios. Housing diversity was similar for all three clusters.

Three case studies were chosen to illustrate differences in the built environment features of each NAC type. Aerial satellite imagery, street frontage views and summary statistics for each case study can be found in the online Additional file 2: Appendix 2 Case Studies.

### Location of NACs

Figure 2 shows the spatial distribution of NACs by cluster type. HW NACs are generally located close to the Central Business District (CBD) with the remaining cluster types distributed according to distance from the CBD. A small number of MW NACs were located in outer urban areas, and a small number of LW NACs were located in middle suburbs.

### Socio-demographic profiles and walking behavior

Table 3 presents the socio-demographic profile of VISTA participants. Those living near HW NACs, were more likely to be studying and living in other household structures. They were also more likely to be working full-time and have incomes exceeding $2500 per week, and are more likely to live in areas with low area-level disadvantage. While there were high proportions of motor vehicle owners in all three clusters, this was lower (81%) for those living near HW NACs.

**Table 3:** Socio-demographic profiles of survey participants across neighbourhood activity centre types

Covariate			Walkability		
		Overall (n=19,984) N (%)	High (HW) (*n*=757) N (%)	Moderate (MW) (*n*=9,259) N (%)	Low (LW) (*n*=9,968) N (%)
Sex	Male	9,516 (47.6)	361 (47.7)	4,344 (46.9)	4,811 (48.3)
	Female	10,468 (52.4)	396 (52.3)	4,915 (53.1)	5.157 (51.7)
Age^a^		46.34 (17.1)	43.13 (15.8)	46.85 (17.6)	46.10 (16.6)
Studying	Yes	2,179 (10.9)	128 (16.9)	1,097 (11.9)	954 (9.6) *
	No	17,805 (89.1)	629 (83.1)	8,162 (88.2)	9.014 (90.4)
Occupation	Unemployed	6,225 (31.2)	194 (25.6)	2,932 (31.7)	3,099 (31.1)*
	Casual work	1,522 ( 7.6)	54 ( 7.1)	751 (8.1)	717 (7.2)
	Part-time	3,053 (15.3)	115 (15.2)	1,405 (15.2)	1,533 (15.1)
	Full-time	9,184 (46.0)	394 (52.1)	4,171 (45.1)	4,619 (46.3)
Household Structure	Couple with children	7,755 (38.8)	437 (57.7)	3,867 (41.8)	3,451 (34.6) *
	Couple without children	10,070 (50.1)	195 (25.8)	4,290 (46.3)	5,585 (56.0)
	Other household structure	2,159 (10.8)	125 (16.5)	1,102 (11.9)	932 (9.4)
Income	<$650	2,387 (11.9)	102 (13.5)	1,194 (12.9)	1,091 (11.0) *
	$650-$1099	3,227 (16.2)	105 (13.9)	1,443 (15.6)	1,679 (16.8)
	$1200-$1949	3,724 (18.6)	125 (16.5)	1,584 (17.1)	2,015 (20.2)
	$1950-$2499	4,357 (21.8)	132 (17.4)	1,890 (20.4)	2,335 (23.4)
	$2500+	6,289 (31.5)	293 (38.7)	3,148 (34.0)	2,848 (28.6)
Own a motor vehicle	Do not own a motor	946 ( 4.7)	139 (18.4)	609 ( 6.6)	198 ( 2.00) *
	Own a motor vehicle	19,038 (95.3)	618 (81.6)	8,650 (93.4)	9,770 (98.0)
Area level disadvantage ^b^	High	5,545 (27.8)	128 (16.9)	2,535 (27.4)	2,882 (28.9) *
	Medium	5,798 (29.0)	124 (16.4)	2,442 (26.4)	3,232 (32.4)
	Low	8,641 (43.2)	505 (66.7)	4,282 (46.3)	3,854 (38.7)

^* ^Statistical significance between clusters assessed using chi-squared tests for categorical variables and ANOVA for continuous variables: *p*<0.05

^a ^Means and standard deviations presented

^b ^Based on SEIFA IRSD deciles where 1-4 indicate higher levels of area based disadvantage, 5-7 is medium and 8-10 is low

There were many similarities in the socio-demographic profile of people living near MW and LW NACs. Participants living near these two areas were similar in terms of occupation and area level disadvantage, with almost one third unemployed, and around two thirds living in areas with high or medium disadvantage. Notably, participants living near LW NACs were less likely to be studying and more likely to be a couple with children compared with participants living near MW NACs. Almost 100% of participants living near LW NACs owned a motor vehicle (98%).

A descriptive analysis of walking behavior showed that 22.1% or 4,412 participants undertook *any transport walking* and 16.7% or 3,339 participants undertook *any neighbourhood transport walking*.

### Association between NAC type and transport walking trips

Table 4 presents descriptive statistics for road network distances to NACs. The mean distance for people living near HW NACs was 598.8m (SD: 335.4m). The descriptive statistics indicate that overall 76% of survey participants had their closest NAC within 1.6km; 44% were within 1km; and, 29% were within 800m. For people living near HW NACs, 88% lived within 1km, which meets Victoria’s policy requirement that “80-90% of households should be within 1km of an activity centre of sufficient size to allow for provision of a supermarket” [42].

**Table 4:** Descriptive statistics on the distance to the closest neighbourhood activity centre by type

Neighbourhood Activity Centre	Obs^a^	Mean	SD^a^	Min	Max	Obs ≤1.6km	% ≤1.6km	Obs ≤ 1km	% ≤ 1km	Obs ≤ 800m	% ≤ 800m
High (HW)	757	598.8	335.4	27.0	1,826.6	754	99.6	665	87.9	548	72.4
Moderate (MW)	9,259	1,002.6	589.3	2.8	5,162.3	8001	86.4	5,368	58.0	4005	43.3
Low (LW)	9,968	1,459.6	738.9	75.1	5,042.1	6383	64.0	2,752	27.6	1736	17.4
Overall	19,984	1,215.3	708.9	2.8	5,162.3	15,138	75.8	8,785	44.0	5741	28.7

^a^ *Obs* Number of observations, *SD* Standard deviation

**Table 5:** Logistic regression results of any transport walking and any neighbourhood transport walking by neighbourhood activity centre type (*n*=19,984) ^a^

Neighbourhood Activity Centre	Any transport walking OR (95% CI)^b^	Any neighbourhood transport walking OR (95% CI)
Low (LW)	ref	ref
Moderate (MW)	2.28 (1.96, 2.66)^***^	2.75 (2.26, 3.33)^***^
High (HW)	5.85 (4.22, 8.11)^***^	8.66 (5.89, 12.72)^***^

^***^ *p*<0.001

^a ^Models adjusted for the following confounders: sex, age, studying, household structure, income, motor vehicle ownership, area level disadvantage. Models estimated using 3 level multilevel logistic regression according to SA2, household and individual levels

^b^ *OR* Odds ratios, *CI* Confidence intervals

Multilevel logistic regression results showed that compared with those living near LW NACs, those located near HW and MW NACs had increased odds of undertaking 1 or more transport walking trips (HW, OR: 5.85, CI: 4.22, 8.11 and MW, OR: 2.28, CI: 1.96, 2.66) and 1 or more neighbourhood transport walking trips (HW, OR: 8.66, CI: 5.89, 12.72 and MW, OR: 2.75, CI: 2.26, 3.33) (Table 5).

## Discussion and conclusion

### Cluster findings

Clustering NACs by their built environment features produced three clusters characterized empirically by their level of walkability consistent with previous research [4, 37]. The NAC cluster typologies were generally located in inner, middle and outer areas of Melbourne, which, although not analyzed here, relates to their period of construction from the 1800s through to the 21^st^ century. Overall, more walkable NACs were located in older neighbourhoods.

### Summary and discussion of NAC built environment cluster results

HW NACs were characterized by pedestrian-friendly community design, movement network and lot layout. However, contrary to what might be expected, in Melbourne, HW NACs also had high cul de sac values - which have been shown to detract from the walkability of an area [37, 57, 58]. This appears to be because streets in Melbourne’s inner city were designed with rear laneway access for household services and sewerage collection that now provide access to subdivided blocks and small studio style apartments. Furthermore, many inner city streets have been blocked to encourage traffic flows on nearby arterial roads instead of on residential streets. Indeed, more high traffic volume roads were found in HW NACs as evidenced by the low traffic exposure ratio. HW NACs tended to be located on arterial ‘main street’ style shopping strips that house a diversity of shops, services, and high quality public transport, which together have been associated with transport walking [23]. Typically, gridded street networks sit behind these arterial streets providing easy pedestrian access, which is consistent with the notion of transit oriented development [59].

The MW NACs retained many, but not all, of these pedestrian-friendly built environment features with the lowest values for cul de sacs and cul de sac segments≤120m long, connected and disconnected node ratios. This suggests that whilst these areas are walkable and encourage transport walking, they are also conducive to driving, with the streets retaining an open grid structure unhampered by cul de sacs.

The LW NACs had the least pedestrian-friendly design and were more spatially dispersed across the outer suburbs with greater distance to, and between NACs leading to potential inequity in access to local living destinations, employment opportunities, and public transport for residents in these areas. The summary statistics for the built environment features support the low walkability of these NACs with the curvi-linear street structure a likely contributor. The LW NACs showed large tracts of land dedicated to car parking, which has been shown to be a barrier for walking and a design that privileges motor vehicle travel as a means of getting to NACs and other destinations [60–62] (see online Additional file 2: Appendix 2 Case Studies).

Finally, regardless of NAC typology there appeared to be limited differences in housing diversity with approximately 6-7 different types of housing available. To some extent, similarities in housing diversity was expected, since Melbourne is a large homogenous low density sprawling city, particularly in the middle and outer suburbs where detached housing is prominent and where infill density is only now starting to occur [63]. Policy and practice could facilitate the delivery of greater housing diversity to suit different housing requirements across the life course facilitating ageing in place [64]. Increased housing diversity is also a good way to increase dwelling density, which supports walkability by making public transport, services and retail more viable. It also helps to deliver the 20 minute city[63, 65]. However, at least in Australia, current planning policy focuses mainly on increasing densities in inner and middle areas with less emphasis on outer areas. Yet low density development in outer suburban areas not only fosters motor vehicle dependencies, but reduces access to the early delivery of public transport, shops and services. Hence these areas need the most planning and policy support,[63], since many studies now show that where we live can affect our health [66–68] and access to services is a social determinant of health [69]. Furthermore, the metrics for LW NACs show low transport diversity and low numbers of transport stops compounding the disadvantage experienced in these areas, since residents have little choice but to purchase at least one motor vehicle in order to access jobs and essential services [70]. This is supported by our results, showing a correlation between area level disadvantage and the presence of either MW or LW NACs suggesting that there is some socio-spatial patterning and disparities in the provision and type of NAC across middle and outer suburbs. This finding also aligns with the lack of policy implementation which we consider further below.

### Are different NAC types associated with transport walking?

Multilevel logistic regression models assessing the influence of the three NAC types on transport walking showed a significant trend of increased odds of any transport walking and any neighbourhood transport walking corresponding to the more walkable NACs. Those living near HW NACs had odds 6-9 times higher for walking for transport than those living near LW NACs. Like other authors [4, 29, 71, 72], these results suggest that built environment features have considerable influence on transport walking behavior in providing both access and destinations to walk to [27, 29, 59, 73].

### Planning policy implementation of supermarket provision

Few studies objectively evaluate the implementation of urban design and planning policies [43]. Hence, we assessed the current state government policy requirement that “80-90% of households should be within 1km of a town centre of sufficient size to allow for provision of a supermarket.” We found that only households located near HW NACs met the policy requirement at a threshold of 1km (88%). Indeed, only 28% of households with LW NACS met this policy requirement. This highlights discrepancies between current policy requirements and policy implementation, especially for those living in outer suburban areas where NACs are spatially dispersed, suggesting inequitable delivery of this policy requirement across the city.

To support walking and population health there needs to be a greater focus on adherence and implementation of policy requirements [43], especially since many policies are non-binding, as is the case here. Our findings provide some evidence to support governments to strengthen policy requirements for reducing the distance between residences and NACs; and to support greater efforts in evaluating policy implementation in practice. These findings provide a “call to action” to provide well designed areas that support people and communities. This is important since it is difficult to retrofit neighbourhoods to build NACs or to provide other important infrastructure such as parks and open spaces, public transport, and schools without ensuring adequate land is allocated for these purposes in the land use planning phase of a housing development.

### Strengths and Limitations

The focus on objective measures of the built environment surrounding NACs is a strength of this paper. It contributes to emergent evidence of the importance of other environmental exposures beyond where we live for influencing walking behaviors [74–76], which may differ for different types of participants [77, 78]. Another strength, is the presentation of metrics of the built environment surrounding NACs. These are presented for several built environment features, which could inform future urban design guidelines. These metrics could also be used to evaluate and monitor existing and proposed developments and for retrofitting LW NACs to the standards set by MW or HW NACs where appropriate. The provision of metrics for individual built environment features *and* the results of the cluster analysis examining several features jointly are also strengths of this paper, since it is important to understand the types, quantities and mix of built environment features [40, 79].

However, limitations of this analysis include using the road network for GIS calculations of street connectivity and cul de sacs, which may ignore other networks accessible to pedestrians and cyclists. This may under-estimate the values for these built environment variables. Similarly, other built environment features could be added to the analysis, such as the number of transport routes, length of cycleways or, the size or the period of construction of the NAC. This may alter the cluster solution. We did not find any associations with housing diversity. It is possible that our housing diversity variable was not sensitive enough to pick-up variation in housing types. Future research may seek to refine this variable by incorporating the area of lots as well as the type and size of housing.

In this study, we used current local planning documents *Plan Melbourne* to define and inform the identification of NACs. The method adopted may have missed some NACs. The analysis focused on the area surrounding the NAC and measured associations with transport walking trips made by survey participants’ in their home neighbourhoods measured by a 1.6km network buffer. It is possible that survey participants’ undertook transport walking trips in other areas (e.g., near work) and to NACs other than the closest one, as was assumed in this study. We used cross-sectional data and hence, do not know the temporality of associations and consequently we could not assess whether socio-spatial disparities and differences in transport walking were driven by self-selection or social inequity or a combination of both. However, no information on neighborhood preferences was collected in the VISTA 2009-2010 survey and whilst more recent VISTA data is available a temporal mismatch between the survey and GIS data negated its use. Issues, like these, relating to deprivation amplification [66] in service provision and transportation across Melbourne are complex and are left for future work. The labeling of the cluster typologies according to levels of walkability (e.g. high, moderate, low) was empirically driven and relates to the context of Melbourne, Australia. We recommend that future research place such findings within the international context aligned to emergent evidence on thresholds for built environment variables [10]. Nevertheless, the findings here show that certain levels for the types, quantities and mix of built environment features are required to support transport walking. Finally, the dependent variables were based on one survey day measuring transport walking behavior. As such, the two definitions of transport walking used here were insufficient for determining whether or not survey participants undertook adequate physical activity.

## Conclusion

This study provides evidence on the type, quantity and mix of built environment features that create walkable NACs. Results show an inequitable spatial distribution across metropolitan Melbourne, with HW NACs generally located in inner areas, and LW NACs in outer areas. Furthermore, LW NACs in outer areas were less likely to meet policy guidelines on supermarket provisioning, with these areas providing fewer active transport options and resulting in lower levels of transport walking. Overall, the results suggest that both the built environment features and dwelling density surrounding NACs play a key role in influencing travel behavior and in particular transport walking.

Features of HW NACs that encourage walking include: high street connectivity (mean:161), high destination diversity (mean:16), and high residential density (mean:77). These metrics are significantly higher than LW NACs characterized by significantly lower street connectivity (mean:57), destination diversity (mean:11), and residential density (mean:10). Identifying and providing summary statistics and metrics on a broad range of built environment features associated with increased walking is requested by decision makers [19], and will support them to develop planning policy and urban design guidelines that create healthy, more equitable and sustainable communities. This moves beyond the rhetoric of the need for more walkable communities, to supplying metrics on *how* this can be achieved. More research of this type in different cities is warranted.

## Additional files

Additional file 1: Appendix 1 VISTA Sample. (DOCX 18 kb)

Additional file 2:. Appendix 2 Case Studies. (DOCX 1158 kb)

## Abbreviations

CBD: Central Business District
GIS: Geographic Information Systems
HW: Highly Walkable
LW: Low Walkable
MW: Moderately Walkable
NACs: Neighbourhood Activity Centres
VISTA: Victorian Integrated Survey of Travel Activity
