# Supplementary material for: Designing healthy communities: creating evidence on metrics for built environment features associated with walkable neighbourhood activity centres
Source: Int J Behav Nutr Phys Act. 2017 Dec 4;14:164. doi: 10.1186/s12966-017-0621-9 (PMC5716232; doi:10.1186/s12966-017-0621-9)
Supplement: Supplementary file 1 — Appendix 1 VISTA Sample. (DOCX 18 kb) [file 12966_2017_621_MOESM1_ESM.docx]

Additional File 1 – VISTA Sample

The VISTA survey was conducted by The Victorian Department of Transport (now known as the Victorian Department of Economic Development, Jobs, Transport, and Resources) on a target population including metropolitan Melbourne and five Victorian regional areas for one day of travel behaviour. Only metropolitan Melbourne was used in this analysis.

A stratified multi-stage sampling method was employed sampling Census Collector Districts (CCDs) first followed by households. Census Collector Districts are part of the Australian Geographical Standard (ASGC) applicable to the 2006 census and the VISTA survey. CCDs cover on average 225 dwellings. The sampling method was based on the VISTA 2007 survey with metropolitan Melbourne divided into 8 regions with an approximately equal number of households selected from each area to ensure an equal probability of selection. The proposed method was adjusted to allow for differences in the response rates and to ensure that approximately equal numbers of participants were selected across regions. Despite this for some of the smaller regions sample sizes were smaller than anticipated due to low survey response rates, as such the survey data is not recommended for regional spatial analyses at the local government level. This is not an issue for the study conducted here, as it is based on smaller spatial units based on the Statistical Area 2 (SA2) which accounted for the clustering of households within the VISTA survey. SA2 areas cover approximately 10,000 households. In conducting the survey no additional biases were reported by the Victorian Department of Economic Development, Jobs, Transport, and Resources.

The study is based on a subset of the full sample corresponding to adults aged over 18 and is based on a complete case analysis. In preparing the data several participants were removed due to a lack of data detailing their area level disadvantage (n=155) and a further 7% of the sample were not associated with a neighbourhood activity centre due to living on the extreme periphery of the city. Due to the large sample size, we did not expect these adjustments to have a large impact on the results.
