# Supplementary material for: Designing healthy communities: creating evidence on metrics for built environment features associated with walkable neighbourhood activity centres
Source: Int J Behav Nutr Phys Act. 2017 Dec 4;14:164. doi: 10.1186/s12966-017-0621-9 (PMC5716232; doi:10.1186/s12966-017-0621-9)
Supplement: Supplementary file 2 — Appendix 2 Case Studies. (DOCX 1158 kb) [file 12966_2017_621_MOESM2_ESM.docx]

Appendix – Case studies of Neighbourhood Activity Centres (NACs)

Additional detail and information for each of the case studies including aerial imagery is presented in this Appendix. Aerial satellite imagery was obtained from Google Maps, with Google Imagery and Map data@2016.

*Case Studies of NAC*

Three case studies were chosen to illustrate differences in the built environment features of a representative NAC for each cluster. Since the anchor for each NAC was a supermarket, the supermarket address was used to help identify each case study. For brevity, the case study NACs are referred to by their suburb name. Figure A1 displays aerial satellite imagery, and street frontage views for each case study with summary statistics presented in Table A1.

*Case Study 1:* *Highly Walkable Activity Centre – Fitzroy*

This supermarket is located in Melbourne’s first suburb of Fitzroy established in 1889, located 2km north east of Melbourne’s central business district (CBD) ([Internet Archive WayBack Machine](#_ENREF_28)). It is located near the main commercial district of Brunswick Street which is currently a vibrant retail zone with many restaurants, cafés and clothing stores. The area was originally working class and was planned and constructed to allow blue collar workers to walk to nearby flour mills, footwear and clothing factories. In 1886 three tramlines were opened along the main access routes from the city of Nicholson, Brunswick and Smith Streets. The Nicholson and Smith Street trams remain some of Melbourne’s busiest routes today ([Yarra Trams](#_ENREF_57)). Several social housing estates were constructed from the 1950s, and despite gentrification in recent times, the area retains broad socioeconomic and ethnic diversity in part due to the social housing. Today, conversion of old factories and warehouses to apartments has increased Fitzroy’s residential density.

Figure A1a shows an aerial photo surrounding the supermarket and NAC in Fitzroy. The photo shows large retail commercial areas along Nicholson, Johnston and Brunswick Streets and some vertical residential apartments in addition to original Victorian terrace housing. The area follows a street grid system characterized by small average square shaped block sizes (mean: 366.11m) with high street connectivity (mean: 154.18). This area has a high number of destinations regardless of destination type, and has high destination diversity (mean: 16). The area has both high transport accessibility (mean: 3), net residential density (mean: 46.7) and high numbers of cul de sacs (mean: 182) and cul de sac segments≤120m (mean: 173).

*Case Study 2: Moderately Walkable – Reservoir*

Reservoir is located 12km north of Melbourne’s CBD and is an area first established around 1864. It remained rural until post World War II when residential development was rapid with two primary schools and a high school being constructed in the early to mid-1950s, and another two primary schools and a high school constructed throughout the 1960s. The NAC and supermarket in Reservoir located on Spring Street opposite the Reservoir train station have been in existence since the 1920s ([Internet Archive WayBack Machine](#_ENREF_28)).

Figure A1b shows the Reservoir NAC. The NAC is a main street style commercial zone that contains well established shops on two perpendicular streets: Spring Street and Edwardes Street. The area is well served with public transport with a train station and numerous bus stops in the vicinity (mean: 99). The area has predominantly single story detached housing although some infill development and apartments are currently being constructed. Specifically the aerial photo shows that housing blocks are larger than those in Fitzroy. Whilst it retains a gridded street network, the aerial photo shows that these blocks are long and rectangular and on average approximately twice the size of those in the Fitzroy example (mean: 716.8m) corresponding with lower street connectivity (mean: 52.93). Street accessibility is greater than both Fitzroy and South Morang (see Case Study 3) with low numbers of cul de sacs (mean: 21) and cul de sac segments≤120m (mean: 14). Whilst there are less destinations present than the Fitzroy NAC, the destination diversity is similar (mean: 14). Several small car parks behind Edwardes and Spring Streets are evidence of the shift in transport mode to car orientated access. The traffic exposure ratio is 0.81, which suggests that the area is surrounded by residential streets with few high traffic volume arterial roads.

*Case Study 3: Low Walkable – South Morang*

South Morang is located 25km north-east of Melbourne’s central business district along the Plenty river. It has become a relatively new residential area since its construction commencing in the late 1990s ([Internet Archive WayBack Machine](#_ENREF_28)). The area contains residential areas and large tracts of parkland and nature reserves including the Plenty Gorge, Hawkstone and Granite Hills Parks. In 2012, the Epping train line was extended to South Morang.

Figure A1c shows an aerial photo of the Rivergum Shopping Centre in South Morang. The area is characterized by large scale retail including fast food stores, a hardware and timber warehouse, and a car dealership. The aerial photo shows a high proportion of area dedicated to car parking with the various retail and shopping zones dissected by Plenty Road which is the main traffic arterial for this area. Compared to Fitzroy and Reservoir, net residential density is low in this area (mean: 10.25) with large single family homes evident in Figure A1c. This area has lower destination diversity (mean: 12), and compared to the other two case studies, lower numbers of destinations by destination type. The only form of public transport are buses, with a total of 39 bus stops in the area. Street connectivity is slightly higher than the Reservoir case study, however Figure A1c shows that the street pattern is curvi-linear. Of the three case studies, block sizes (mean: 3259.33m) and disconnected node ratios (mean: 1.70) are highest for South Morang and values for cul de sacs and cul de sac segments≤120m sit between the values presented for Fitzroy and Reservoir.

*Summary of Case Studies*

The three case studies provide specific examples of NAC built environment features identified by the three cluster types. The Fitzroy example is highly walkable with the highest variety of destinations, net residential density and public transport access. Although Reservoir was a MW NAC and retains many of the built environment features of more walkable areas, it allows for car access and is less walkable, as the summary statistics suggest a decrease in overall accessibility relating to street connectivity, block sizes, walkable block ratios and traffic exposure. Finally, South Morang is designed to be car orientated and has low walkability as evidenced by the low net residential density, large block sizes, lower destination diversity, large area dedicated to parking and high traffic volume roads.

Table A1 shows the decline in the percentage of participants undertaking any transport walking trips from the HW Fitzroy area (50%), to the MW Reservoir area (26%), to the LW area of South Morang (0.06%). The percentage is even lower for neighbourhood transport walking trips i.e., HW Fitzroy (42%), MW Reservoir (22%), LW South Morang (0.03%).


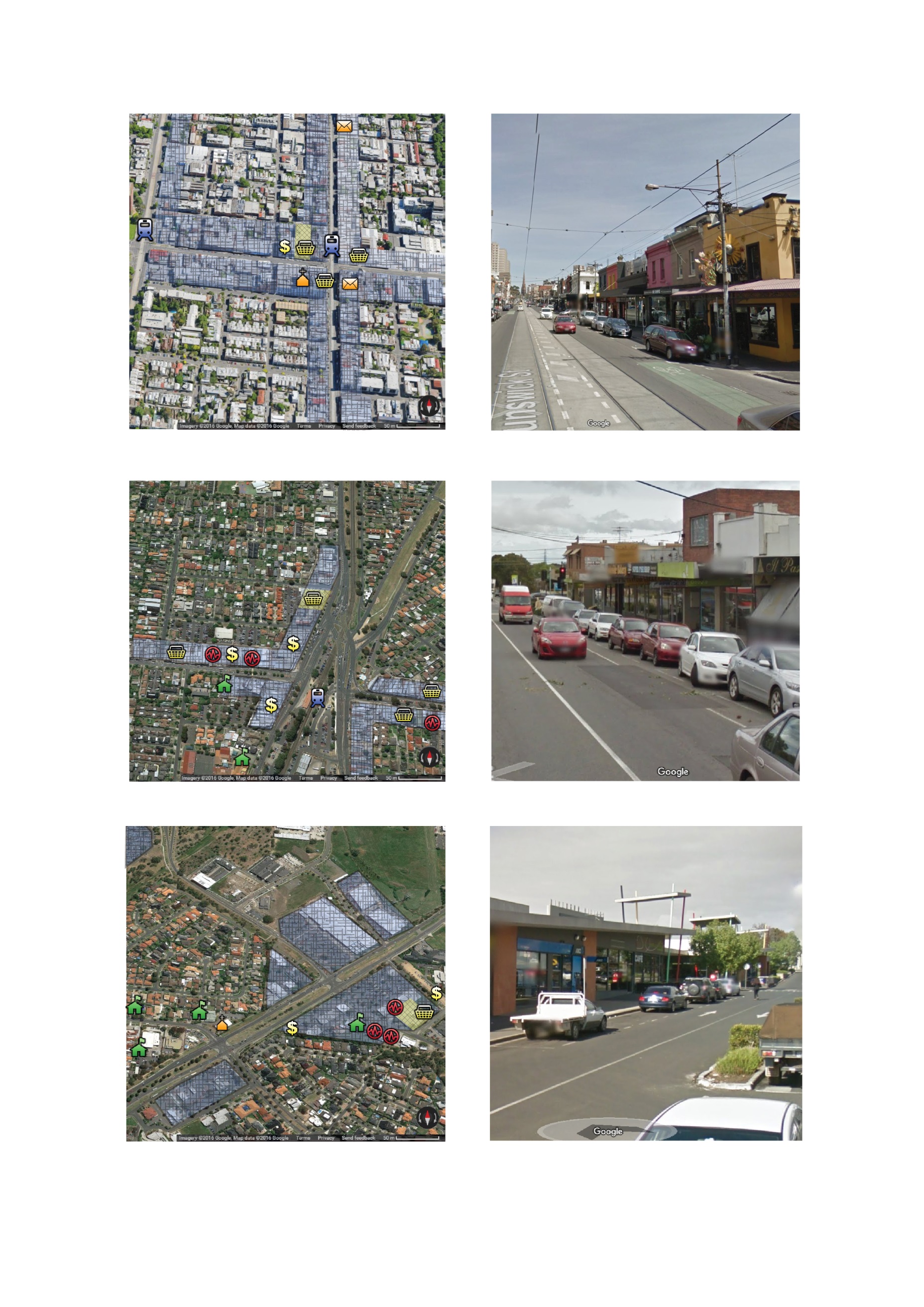


**b) Aerial and street view of a moderately walkable neighbourhood activity centre, Reservoir**

**a) Aerial and street view of a highly walkable neighbourhood activity centre, Fitzroy**

**c) Aerial and street view of a low walkable neighbourhood activity centre, South Morang**

**Figure A1. Aerial and street views of neighbourhood activity centres (NACs)**


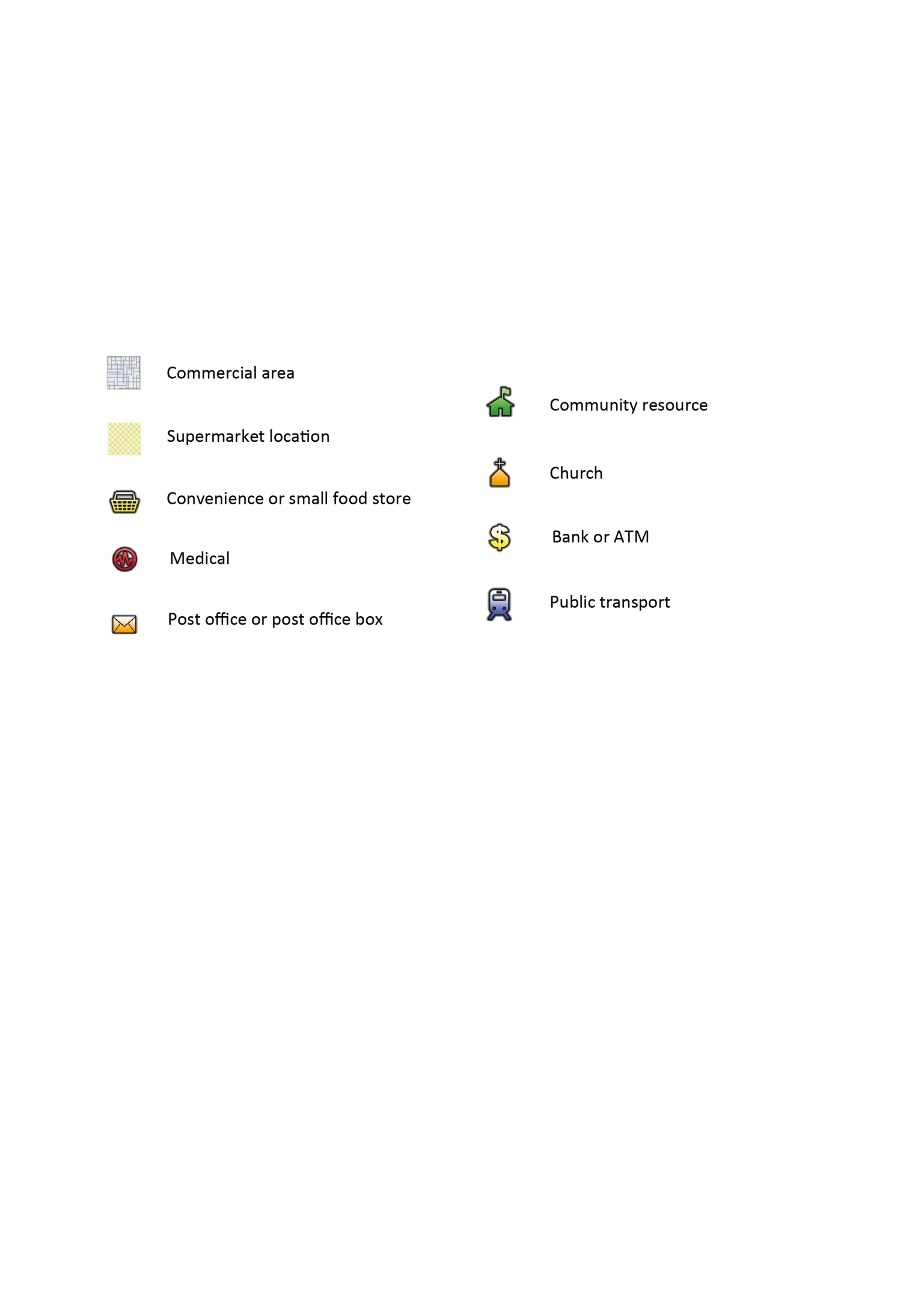


**Figure A1 continued. Key to symbols**

Table A1. Summary statistics for the three case study NACs

|  |  | Walkability |  |
| --- | --- | --- | --- |
|  | High (HW) | Moderate (MW) | Low (LW) |
| Suburb | Fitzroy | Reservoir | South Morang |
| Population^a^ | 9,430 | 47,637 | 20,873 |
| Area^a^ | 1.4km^2^ | 19.1km^2^ | 21.5km^2^ |
| *Walking Trips ^b^* |  |  |  |
| Number of Participants | n=24 | n=73 | n=80 |
| Any Transport Walking (%) | 50.00% | 26.00% | 0.06% |
| Neighborhood Transport Walking (%) | 41.67% | 21.55% | 0.03% |
| *Community Design* |  |  |  |
| Pedshed | 0.68 | 0.66 | 0.43 |
| Community resources | 36 | 13 | 7 |
| Small food stores | 40 | 14 | 3 |
| Other retail | 53 | 17 | 9 |
| Supermarkets | 7 | 2 | 1 |
| Supermarket diversity | 1 | 1 | 1 |
| Transport | 161 | 99 | 39 |
| Transport diversity | 3 | 2 | 1 |
| Destination diversity | 16 | 14 | 12 |
| *Movement Network* |  |  |  |
| Street connectivity | 154.18 | 52.93 | 61.18 |
| Cul de sacs | 182 | 21 | 104 |
| Cul de sac segments≤120m | 173 | 14 | 83 |
| Connected node ratio | 0.18 | 0.07 | 0.33 |
| Disconnected node ratio | 1.18 | 0.40 | 1.70 |
| Mean block perimeter | 366.11 | 716.87 | 3259.33 |
| Walkable block ratio | 0.87 | 0.54 | 0.45 |
| Traffic exposure ratio | 0.75 | 0.81 | 0.76 |
| *Lot Layout* |  |  |  |
| Housing diversity | 6 | 5 | 8 |
| Net residential density | 46.74 | 15.98 | 10.25 |

^a^ Australian Bureau of Statistics (2011). Census of Population and Housing, Basic Community Profile: Fitzroy, Reservoir, South Morang.

^b^ Calculated based on VISTA participants associated with each of the respective NACs undertaking at least one walking trip.
